# Socioeconomic Inequalities in SARS-CoV-2 Infection and COVID-19 Health Outcomes in Urban Italy During the COVID-19 Vaccine Rollout, January–November 2021

**DOI:** 10.1007/s11524-024-00844-0

**Published:** 2024-03-18

**Authors:** Emmanouil Alexandros Fotakis, Alberto Mateo-Urdiales, Massimo Fabiani, Chiara Sacco, Daniele Petrone, Flavia Riccardo, Antonino Bella, Patrizio Pezzotti

**Affiliations:** 1 European Programme On Intervention Epidemiology Training (EPIET), European Centre for Disease Prevention and Control, Stockholm, Sweden; 2 Department of Infectious Diseases, Istituto Superiore Di Sanità, Rome, Italy

**Keywords:** COVID-19 vaccination, Health inequalities, Socioeconomic deprivation

## Abstract

This study analysed the evolution of the association of socioeconomic deprivation (SED) with SARS-CoV-2 infection and COVID-19 outcomes in urban Italy during the vaccine rollout in 2021. We conducted a retrospective cohort analysis between January and November 2021, comprising of 16,044,530 individuals aged ≥ 20 years, by linking national COVID-19 surveillance system data to the Italian SED index calculated at census block level. We estimated incidence rate ratios (IRRs) of infection and severe COVID-19 outcomes by SED tercile relative to the least deprived tercile, over three periods defined as low (0–10%); intermediate (> 10–60%) and high (> 60–74%) vaccination coverage. We found patterns of increasing relative socioeconomic inequalities in infection, hospitalisation and death as COVID-19 vaccination coverage increased. Between the low and high coverage periods, IRRs for the most deprived areas increased from 1.09 (95%CI 1.03–1.15) to 1.28 (95%CI 1.21–1.37) for infection; 1.48 (95%CI 1.36–1.61) to 2.02 (95%CI 1.82–2.25) for hospitalisation and 1.57 (95%CI 1.36–1.80) to 1.89 (95%CI 1.53–2.34) for death. Deprived populations in urban Italy should be considered as vulnerable groups in future pandemic preparedness plans to respond to COVID-19 in particular during mass vaccination roll out phases with gradual lifting of social distancing measures.

**Supplementary Information:**

The online version contains supplementary material available at 10.1007/s11524-024-00844-0.

## Introduction

The COVID-19 pandemic has profoundly impacted our societies and continues to pose an important public health threat worldwide. In Italy, as of the end of 2023, COVID-19 has caused more than 804,000 hospitalisations and 192,000 deaths since February 2020, when the first autochthonous case was notified [1] whilst largely affecting the country’s economy and urban social fabric.

Contrary to the initial perception that the pandemic affected all social classes equally [2], international literature shows substantial socioeconomic inequalities in SARS-COV-2 infection, COVID-19 hospitalisation and COVID-19 death, with socioeconomically disadvantaged populations being disproportionally affected [3, 4].

Several conceptual frameworks theorise the driving force of this unequal COVID-19 experience in pre-existing inequalities in the prevalence of chronic diseases and the social determinants of health [5, 6]. In addition, several studies show that socioeconomic inequalities in COVID-19-related outcomes do not appear static over time nor uniform in space but instead may reproduce or change in response to changing epidemic dynamics and different public health policy factors [4].

Indicatively, studies from Italy and other high income countries show that the lockdown policies implemented during the early stages of the pandemic often coincided with increased or inverted inequalities in SARS-CoV-2 infection, predominantly to the disadvantage of more deprived populations [4, 7]. In particular, it is widely proposed that the elevated relative risk in those experiencing socioeconomic deprivation likely resulted from increased viral exposure and transmission, mediated by the increased barriers experienced by these populations in adhering to physical distancing, owing to their living and working conditions [6].

As of early 2021, vaccination gradually became the principal tool to mitigate the impact of the COVID-19 pandemic. In Italy, the COVID-19 vaccine rollout started on December 27, 2020, reaching a 70% vaccination coverage of at least one vaccine dose by mid-September 2021 [8], by which time Public Health and Social Measures (PHSMs) (complementing the vaccine centred pandemic response) had largely been lifted. Notably, the study conducted by Sacco and colleagues in Italy, shows that COVID-19 vaccination substantially altered the course of the pandemic in the country, averting more than an estimated 79,000 COVID-19 hospitalisations and 22,000 deaths, in the first nine months of 2021 [9].

However, information on how socioeconomic inequalities in SARS-CoV-2 infection and COVID-19 health outcomes evolved during the COVID-19 vaccine rollout in Italy and elsewhere remains scarce. Urban settings especially, which have largely been in the epicentre of the COVID-19 pandemic and vaccine rollout campaign efforts in Italy [10] and other countries, exhibit marked social inequalities, intense socioeconomic segregation and high population densities; potentially exacerbating socioeconomic inequalities in COVID-19 health related outcomes [11]. Yet large-scale studies estimating socioeconomic inequalities in COVID-19 health outcomes in urban populations in support of public health policy planning are lacking.

Deciphering the impact of the COVID-19 vaccination rollout on socioeconomic inequalities in COVID-19 health outcomes in urban populations could inform authorities in the planning and implementation of targeted interventions reducing health inequalities in cities, and to guide future pandemic preparedness. We aimed to investigate the associations between socioeconomic deprivation (SED) and SARS-CoV-2 infection and COVID-19-related hospitalisation and death in the adult population of urban Italy; and how these associations changed between periods of increasing COVID-19 vaccination coverage in 2021.

## Methods

### Study Design

We conducted a population-based retrospective cohort analysis using a contextual approach. We measured the associations between SED and the incidence rates of SARS-CoV-2 infection, COVID-19 hospitalisation and COVID-19 death for three consecutive periods with different COVID-19 vaccination coverage of at least one dose of any COVID-19 vaccine type. The three periods analysed were: January 1, 2021 (corresponding to day six of the COVID-19 vaccination rollout in Italy) to March 24, 2021 (at which date the Italian national COVID-19 vaccination coverage was 10%); March 25, 2021 to July 25, 2021 (at which date vaccination coverage was 60%) and July 26, 2021, to November 4, 2021 (at which date vaccination coverage was 74%). The three periods were defined as low, intermediate and high vaccination coverage period respectively.

### Data Sources

We obtained individual data on cases, hospitalisations and deaths from the National COVID-19 Integrated Surveillance System, which collects demographic, clinical and epidemiological data on antigen and/or PCR confirmed cases of COVID-19 in Italy [12]. We retrieved the latest available census estimates of the Italian population stratified by census block, age and sex, corresponding to the year 2011, as well as estimates of SED for each census block area, from ISTAT [13]; and used the revised European Standard Population (EUROSTAT) [14] for population standardisation purposes (supplementary Table S1). Data on the degree of urbanisation of the Italian municipalities (i.e. local administrative units level 2 (LAU2)) and COVID-19 vaccination data were obtained from EUROSTAT [15] and the Italian National Vaccination Registry [8] (extracted on January 14, 2022), respectively.

### Study Population

We included all adults aged 20 years or older, accounting for the 2011 census dataset stratified by 5-year age groups. We then excluded those who did not reside in urban LAU2 areas and those missing information on census block SED. We also excluded population subgroups matched by census block of residence, age and sex, to those with: a SARS-CoV-2 infection prior the study start period; a SARS-CoV-2 infection with missing information on the date of sampling or date of positive testing; and those with a SARS-CoV-2 infection lost during clinical follow-up. The 2011 population estimates were revised by census block, age and sex, by replacing the given values with the number of individuals infected with SARS-CoV-2 when the latter exceeded the respective census estimates.

### Exposure and Outcomes

As a measure of SED (exposure), we used the 2011 Italian social and material deprivation index calculated at the census block level (i.e. the smallest statistical area in Italy) [16]. This index integrates five conditions including unemployment, educational attainment, percentage of rented housing, house overcrowding and percentage of single-parent families. We categorised the index according to terciles of its relative distribution for each urban LAU2 area, with the first tercile (D1) representing the least deprived census blocks and the third tercile (D3) representing the most deprived census blocks.

We measured three outcomes: the incidence rate of notified SARS-CoV-2 infection (asymptomatic or symptomatic); the incidence rate of COVID-19 hospitalisation, defined as a SARS-CoV-2 infection resulting in hospital admission within 28 days and the incidence rate of COVID-19 death, defined as a SARS-CoV-2 infection resulting in death within 28 days.

### Statistical Analysis

The analysis was conducted using surveillance data extracted on December 9, 2021. This allowed the identification of the SARS-CoV-2 infection cases sampled/diagnosed up to November 4, 2021, and their possible COVID-19-related death within 28 days post-infection, considering one week of possible notification delay of the fatal event. We described the baseline characteristics of the study population by SED tercile using counts and percentages.

We then calculated age adjusted weekly incidence rates (IRs) of SARS-CoV-2 infection; COVID-19 hospitalisation; and COVID-19 death, per 1,000,000 person-days, by SED tercile using the revised European Standard Population (EUROSTAT) [14]. For each week, persons were considered at risk for each outcome until the corresponding week-day of sampling/diagnosis of infection or until the last day of the week, whichever came first. Following a similar approach, we calculated cumulative incidence rates for the periods of low, intermediate and high vaccination coverage.

To assess the associations between SED terciles and SARS-CoV-2 infection, COVID-19 hospitalisation and COVID-19 death, we carried out a multivariable analysis using negative binomial regression models for each outcome and calculated incidence rate ratios (IRRs) with 95% confidence intervals (CIs). In all analyses, we used the least deprived tercile (D1) as our reference group and the time of exposure measured in days was included as offset in the model. Participants were considered at risk for infection, hospitalisation and death until the date of sampling/diagnosis of infection or the overall study period end date, whichever occurred first.

The potential confounders included in the models as fixed effects were as follows: sex, region of residence and the interaction between age (categorised into six groups: 20–29, 30–39, 40–49, 50–59, 60–69 and over 70 years old) and vaccination coverage period (i.e. low, intermediate and high vaccination coverage period), assigned after splitting individual data by calendar time. We also considered vaccination coverage period as an effect modifier on the SED—study outcome associations by including the interaction between SED and vaccination coverage period, to measure period specific association estimates within a single model for each outcome. For all models, we used the date of sampling/diagnosis of infection to assign cases, hospitalisations and deaths to one of the three vaccination coverage periods.

Within each vaccination coverage period, the overall association between SED and each outcome was evaluated through the Wald test. For each outcome, the difference in the association with SED amongst vaccination coverage periods was evaluated testing the interaction between SED and period through the likelihood-ratio test.

We conducted both unstratified and stratified multivariable analyses by sex and age group (20–60 years of age and > 60 years of age) to detect potential underlying IRR patterns. Finally, we conducted a sensitivity analysis, by re-estimating our models after splitting individual data into three different periods with corresponding vaccination coverage ranges of 0 to 20%; > 20 to 65% and > 65 to 74%. The analysis was carried out in R (version 4.2.1), using RStudio (version 2022.07.2 + 576).

## Ethical Statement

This study, based on routinely collected data, was not submitted for approval to an ethical committee because the dissemination of COVID-19 surveillance data was authorised by Law number 52 on 19 May 2022 (article 13). Because of the retrospective design and the large size of the population under study, in accordance with the Authorization n. 9 released by the Italian data protection authority on 15 December 2016, the individual informed consent was not requested for the conduction of this study.

## Results

**Fig. 1:**
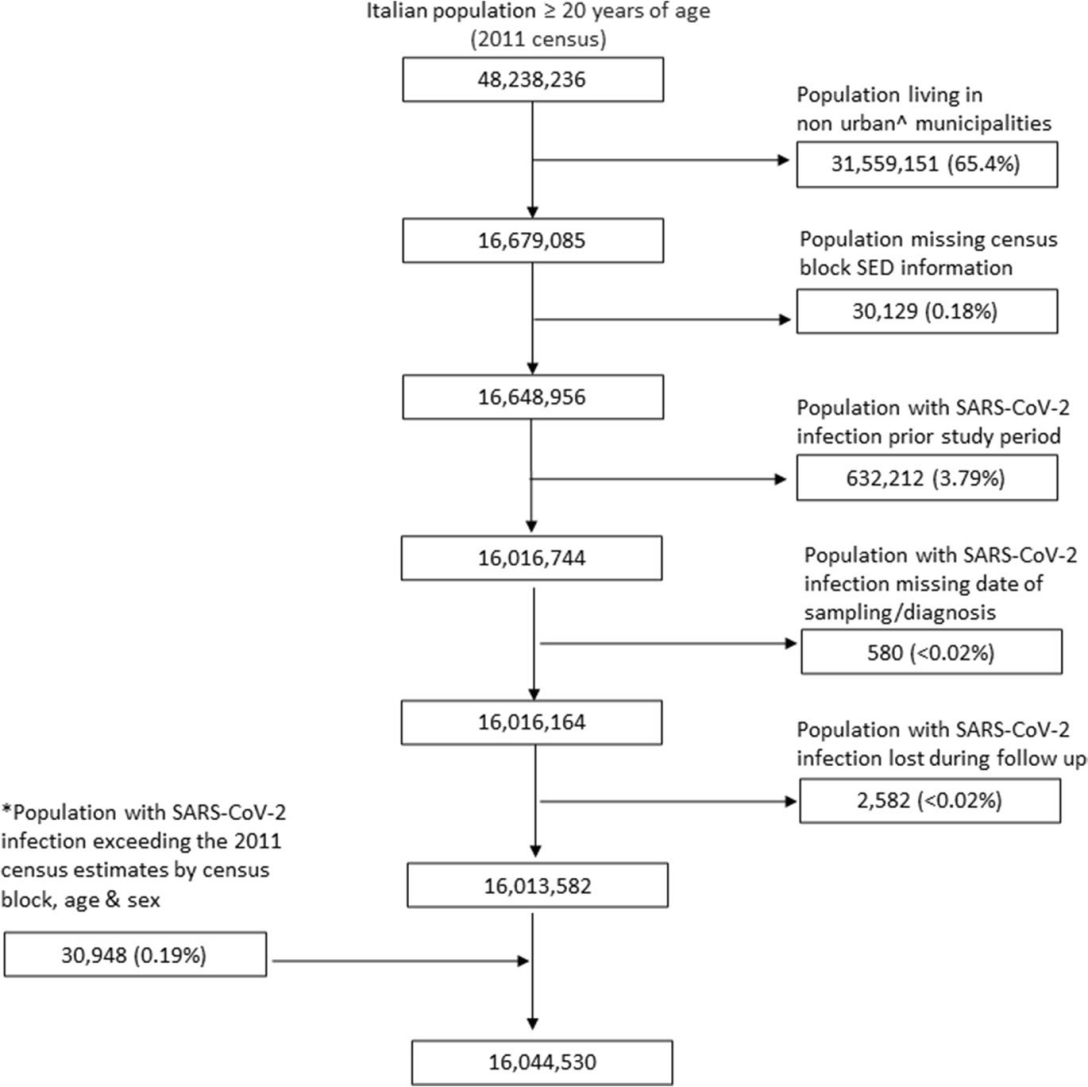
Flowchart showing the inclusion and exclusion criteria for the study population. SED, socioeconomic deprivation. ^Non-urban: intermediate and thinly populated areas (EUROSTAT^15^). *When the 2011 census population estimates by census block, age and sex < population with SARS-CoV-2 infection, the latter values were considered as the respective census block population sizes

We included 16,044,530 persons (Fig. 1) living in 120,341 urban census blocks with a median population size of 75 persons (interquartile range: 30–176). The average follow-up time per participant was 299.5 days. The geographic position of all urban municipalities and census block distribution by SED tercile in the three most populated cities of Italy (i.e. Rome, Naples and Milan) can be found in (supplementary Fig. 1).

The majority of persons (39.9%) lived in D2 census blocks, followed by those (32.4%) in D3 census blocks (i.e. most deprived areas) and (27.7%) in D1 census blocks. The distribution of participants by sex, age group and geographic macro-area was similar between the three terciles (Table 1).

**Table 1:** Study population characteristics; urban Italy, 2021

	D1	D2	D3	Total
Sex				
Female	2,395,275 (54.0%)	3,420,530 (53.4%)	2,755,805 (53.0%)	8,571,610 (52.4%)
Male	2,042,498 (46.0%)	2,986,231 (46.6%)	2,444,191 (47.0%)	7,472,920 (47.6%)
Total	4,437,773 (100.0%)	6,406,761 (100.0%)	5,199,996 (100.0%)	16,044,530 (100.0%)
Age group				
20–29	502,824 (11.3%)	815,774 (12.7%)	738,280 (14.2%)	2,056,878 (12.8%)
30–39	677,205 (15.3%)	1,117,013 (17.4%)	939,914 (18.1%)	2,734,132 (17.0%)
40–49	845,335 (19.0%)	1,300,938 (20.3%)	1,039,558 (20.0%)	3,185,831 (19.9%)
50–59	723,733 (16.3%)	1,035,565 (16.2%)	817,506 (15.7%)	2,576,804 (16.1%)
60–69	717,135 (16.2%)	914,866 (14.3%)	697,756 (13.4%)	2,329,757 (14.5%)
70 +	971,541 (21.9%)	1,222,605 (19.1%)	966,982 (18.6%)	3,161,128 (19.7%)
Total	4,437,773 (100.0%)	6,406,761 (100.0%)	5,199,996 (100.0%)	16,044,530 (100.0%)
Geographic macroarea				
North-West Italy	1,244,888 (28.1%)	1,894,674 (29.6%)	1,509,608 (29.0%)	4,649,170 (29.0%)
South Italy	1,194,908 (26.9%)	1,560,403 (24.4%)	1,227,573 (23.6%)	3,982,884 (24.8%)
Insular Italy	413,333 (9.3%)	619,183 (9.7%)	469,581 (9.0%)	1,502,097 (9.4%)
North-East Italy	605,177 (13.6%)	1,008,466 (15.7%)	874,703 (16.8%)	2,488,346 (15.5%)
Central Italy	979,467 (22.1%)	1,324,035 (20.7%)	1,118,531 (21.5%)	3,422,033 (21.3%)
Total	4,437,773 (100.0%)	6,406,761 (100.0%)	5,199,996 (100.0%)	16,044,530 (100.0%)

D1, D2 and D3 correspond to the least, moderately and most deprived census block areas respectively

### Trends in SARS-CoV-2 Infection and COVID-19 Outcomes According to Socioeconomic Deprivation

**Fig. 2:**
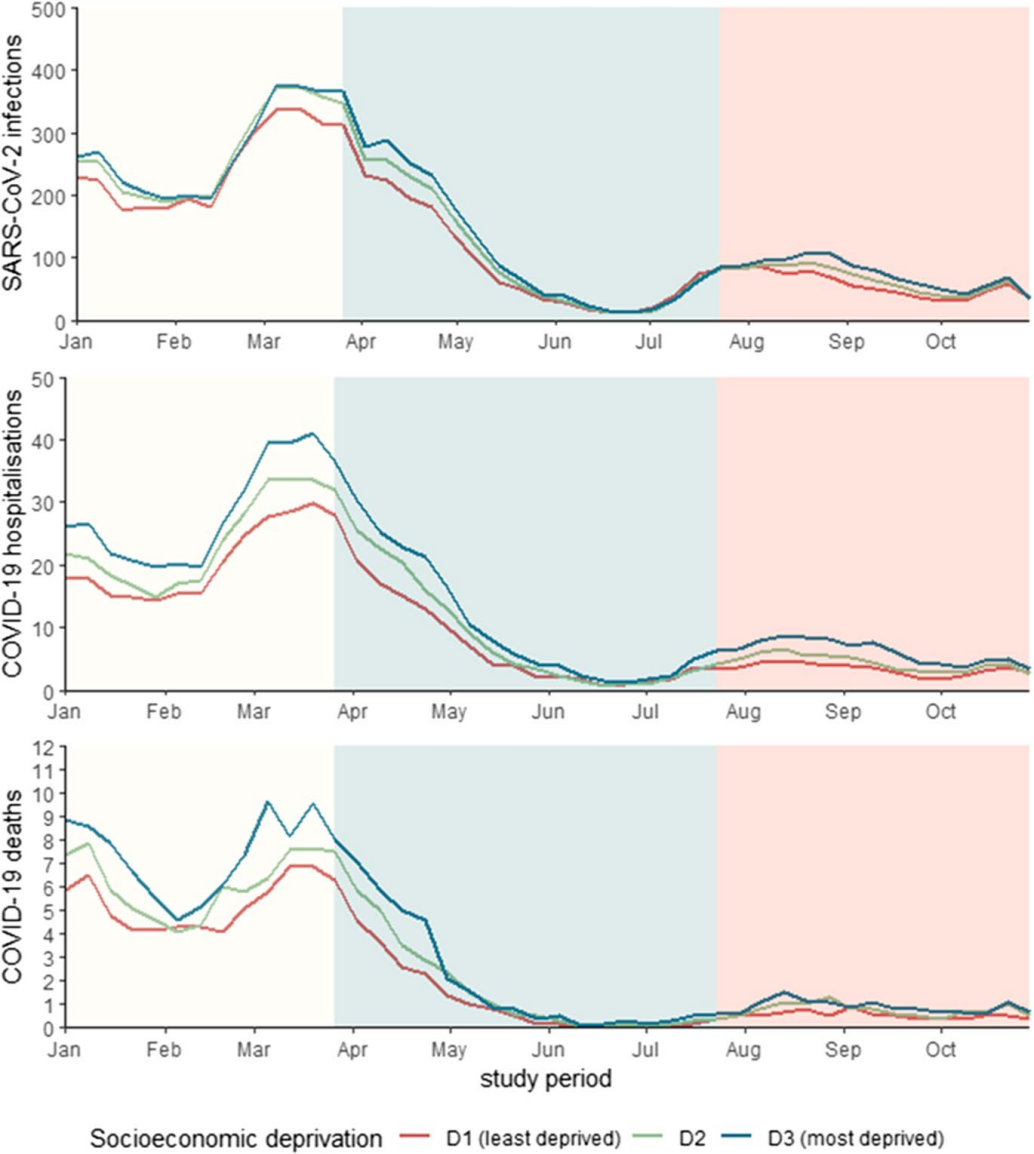
Age adjusted weekly incidence rates of SARS-CoV-2 infection (**A**), COVID-19 hospitalisation (**B**) and COVID-19 death (**C**), per 1,000,000 person-days, by census block socioeconomic deprivation terciles; urban Italy, January-November 2021. Background colours correspond to the three consecutive periods defined as low (0 to 10%), intermediate (> 10 to 60%) and high (> 60 to 74%) vaccination coverage, with at least one COVID-19 vaccine dose. D1 (in red), D2 (in green) and D3 (in blue) correspond to the least, moderately and most deprived census block areas respectively. Age standardisation was conducted using the revised European Standard Population (EUROSTAT) [14]

We observed similar IR trend patterns for SARS-CoV-2 infection, COVID-19 hospitalisation and COVID-19 death (Fig. 2). For SARS-CoV-2 infection and COVID-19 hospitalisation IRs peaked in March 2021. Thereafter, and as vaccination coverage increased, weekly IRs decreased over time followed by a slight increase in late July which thereafter plateaued till the end of the study period. Incidence rates for COVID-19 death followed a similar pattern, yet showed a first peak in early January. Overall, during the low, intermediate and high vaccination coverage periods we observed highest IRs for SARS-CoV-2 infection and both COVID-19 outcomes in D3 areas. The number of events for each outcome and corresponding cumulative IRs by vaccination coverage period are summarised in Supplementary Table 1**.**

### Associations Between Socioeconomic Deprivation and SARS-CoV-2 Infection, COVID-19 Hospitalisation and COVID-19 Death

**Fig. 3:**
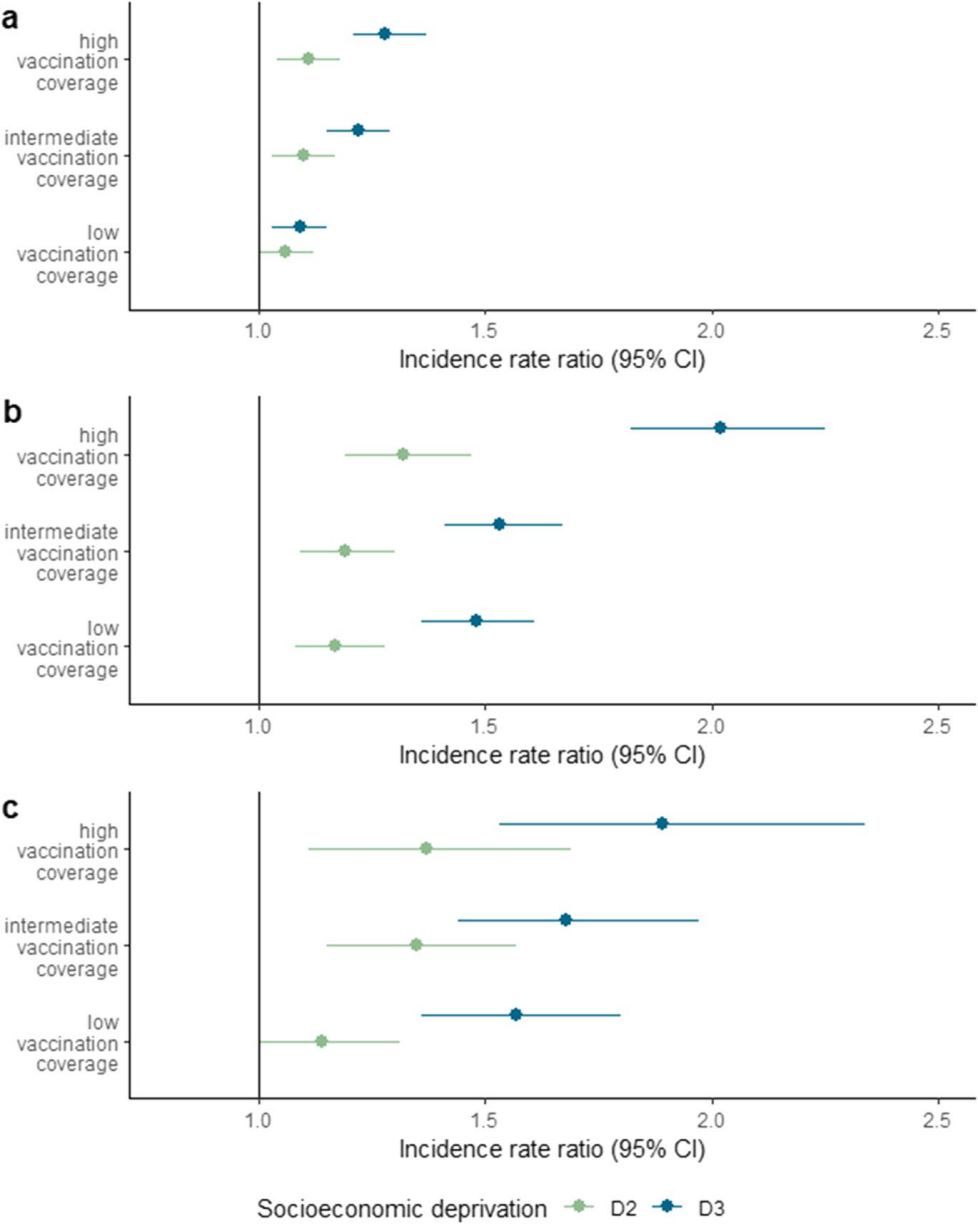
Fully adjusted incidence rate ratios (IRR) of SARS-CoV-2 infection (**A**), COVID-19 hospitalisation (**B**) and COVID-19 death (**C**), by census block socioeconomic deprivation (SED) terciles in urban Italy, and vaccination coverage period. Low vaccination coverage (0 to 10%) / January 1–March 24, 2021; intermediate coverage (> 10 to 60%)/March 25–July 25, 2021; high coverage (> 60 to 74%)/July 26–November 4, 2021, with at least one vaccine dose. The referent tercile (i.e. vertical line at IRR = 1) represents the least deprived areas (i.e. D1 areas). D2 (in green) and D3 (in blue) correspond to the moderately and most deprived census block areas respectively. The models are adjusted for sex, NUTS1 level areas and the interaction between vaccination coverage period and age; with an interaction effect for vaccination coverage period on SED

In each vaccination coverage period, we observed statistically significant patterns of increased IRs of SARS-CoV-2 infection; COVID-19-related hospitalisation and COVID-19-related death, by increasing SED (Wald test; *p* < 0.05). Between vaccination coverage periods (moving from lower to higher coverage), we found patterns of increasing relative socioeconomic inequalities in all study outcomes affecting most, D3 areas (Fig. 3, supplementary Table 2).

For SARS-CoV-2 infection, inter-tercile patterns significantly differed between periods (likelihood ratio test; *p* = 0.003) being more pronounced when vaccination coverage was high. For D3 census blocks which were the most affected areas, IRRs increased from 1.09 (95%CI 1.03–1.15) in the low coverage period to 1.22 (95% CI 1.15–1.29) and 1.28 (95% CI 1.21–1.37) during the intermediate and high coverage periods respectively.

For COVID-19 hospitalisation, inter-tercile patterns also differed significantly between periods (*p* < 0.001) presenting a steeper inter-tercile gradient in the high coverage period, where IRRs peaked. IRRs for D3 census blocks increased from 1.48 (95% CI 1.36–1.61) and 1.53 (95% CI 1.41–1.67) in the low and intermediate coverage periods respectively, to 2.02 (95% 1.82–2.25) when vaccination coverage was high. IRRs also increased for those residing in D2 areas, evolving from 1.17 (95% CI 1.08–1.28) in the low coverage period to 1.32 (95% CI 1.19–1.47) during the high coverage period.

For COVID-19 death, the observed inter-tercile patterns did not significantly differ over time (*p* = 0.39), nonetheless IRRs were again most elevated in the high vaccination coverage period. D3 area IRRs evolved from 1.57 (95% CI 1.36–1.80) in the low coverage period to 1.89 (95% CI 1.53–2.34) when vaccination coverage was high whilst corresponding period estimates for D2 areas were 1.14 (95% CI 1.00–1.31) and 1.37 (95% CI 1.11–1.69).

Our stratified analysis by age did not show marked differences between SED groups, whilst IRRs for SARS-CoV-2 infection and COVID-19 hospitalisation were slightly pronounced amongst females compared to males (supplementary Tables 3, 4). The sensitivity analysis showed similar results to our main analysis (supplementary Table 5).

## Discussion

This is the first study estimating socioeconomic inequalities in SARS-CoV-2 infection, COVID-19 hospitalisation and COVID-19 death in urban Italy over the course of the COVID-19 vaccine rollout in 2021. Our findings, based on data from the whole urban population aged 20 years and over, show that census block-level SED was positively associated with SARS-CoV-2 infection and COVID-19-related hospitalisation and death throughout the vaccine rollout in 2021. Although COVID-19-related incidences reduced across all three SED terciles as the vaccination rollout progressed, we found that socioeconomic inequalities in all study outcomes increased between periods of increasing vaccination coverage.

Overall, the positive associations we found between area level socioeconomic deprivation and COVID-19-related outcomes are in concordance with literature exploring inequalities in COVID-19 prior the vaccine rollout [4]. Similar to our findings, a national scale study conducted in Belgium [17] found increasing socioeconomic inequalities in SARS-CoV-2 infection as vaccination coverage increased. Moreover, in a second study conducted in Madrid, Spain, inequalities in infection affecting deprived areas were shown to peak when vaccination coverage reached 70% of the adult population [18].

Conversely, findings from a study in Bavaria, Germany showed that increasing vaccination coverage appeared to balance incidence and mortality rates between the most and least deprived districts [19]. Two of several factors which could explain these temporal pattern differences are inter-study heterogeneity (e.g. in the study design, study population/setting and measure of deprivation) and cross-country contextual discrepancies (e.g. in socioeconomic factors, the vaccination rollout and the pandemic development).

Existing literature indicates that individuals residing in deprived areas are likely to experience increased SARS-CoV-2 exposure/transmission and elevated vulnerability to severe COVID-19 outcomes, attributed to inequalities in housing, working and pre-existing health conditions [6]. We consider that the interaction of these pathways with vaccine rollout characteristics and several other period-specific factors; differentially affecting socioeconomic groups over the course of the vaccination campaign in Italy, may explain the socioeconomic patterns we observed over time.

We found that socioeconomic inequalities were least pronounced in the early vaccine rollout phase (i.e. vaccination coverage: 0–10%) where eligible groups for vaccination included health care and long-term care professionals, persons ≥ 60 years and individuals with chronic comorbidities [20]. Although the latter population group was prioritised for vaccination, findings from Italy show a higher burden of chronic comorbidities in deprived populations [21] and a positive association between SED and vaccine hesitancy [22]. A seemingly reduced vaccine uptake amongst high risk—socioeconomically deprived populations in the early stages of the vaccine rollout—could contribute to the higher COVID-19-related hospitalisation and death rates we found amongst those residing in more deprived areas, whilst also influencing their susceptibility to infection.

Another distinct characteristic of this “baseline” coverage period was the full implementation of a four-tiered restriction system at the regional level [23]. As previously shown in settings where mobility and social distancing restriction measures are deployed, individuals with manual occupations (primarily residing in more deprived areas) are less likely to be able to work remotely and are overall more likely to suffer from poor working and commuting conditions [5]. These factors, alongside household overcrowding, typically encountered in deprived areas in Italy [16], may have resulted in elevated SARS-CoV-2 exposure and transmission in the more deprived census blocks, explaining the higher infection rates we observed in these areas.

During the intermediate coverage period (i.e. vaccination coverage: > 10–60%; as vaccination became available for people aged ≥ 18 years), we observed that incidence rates for SARS-CoV-2 infection and COVID-19-related hospitalisation and death decreased less in the more deprived areas compared to the least deprived ones, translating to slight IRR increases. In this period restriction measures progressively relaxed after May 2021 [24], and the newly introduced Delta variant gradually supplanted Alpha (which had prevailed over the Ancestral variant during the early vaccination rollout period) [25].

Despite the fact that COVID-19 vaccination in Italy has always been free and with a low threshold access through vaccination hubs, several studies from Italy indicate the emergence of socioeconomic inequalities in COVID-19 vaccination to the disadvantage of deprived populations, as vaccines became increasingly available [26, 27]. Such a reality in conjunction with the successive predominance of variants (i.e. Ancestral to Alpha and then Delta) exhibiting increased infectivity and transmissibility, especially amongst non-vaccinated individuals [28, 29], could account for the inequality shifts we observed across all study outcomes.

Furthermore, despite the relaxation of restriction measures during the intermediate vaccination coverage period, individuals with office/desk jobs, traditionally residing in more affluent areas, largely retained their remote working status as per during the early vaccination rollout phase, whilst manual workers increasingly returned to their on-site work activities. This labour mobility context likely resulted in increased relative infection exposure and subsequent workplace and household transmission in the latter group (primarily residing in deprived areas), contributing to the higher SARS-CoV-2 incidence inequalities we found.

In the high coverage period (i.e. vaccination coverage: > 60–74%), we observed that incidence rates for all study outcomes decreased again less in the more deprived areas compared to the least deprived ones, resulting in enhanced inequalities across all study outcomes. Given the high vaccination coverage during this period (> 70% of the population had a complete primary vaccination cycle by early October 2021 [8]) restriction measures were further reduced [24], whilst Delta was the sole predominant variant [25].

Marked socioeconomic inequalities in vaccination shown to exist in other European countries when vaccination coverage was high [30, 31], especially within a context of high primary vaccination cycle coverage (known to further increase protection against infection and severe disease [32]) could be a key factor influencing the significant IRR increases we observed here, particularly evident for COVID-19 hospitalisation. Likely applicable to the Italian context [26, 27], vaccination inequalities per se, or jointly with the sole predominance of the more virulent Delta variant may have increased the impact of chronic health conditions on severe COVID-19 outcomes amongst unvaccinated/partially vaccinated individuals in the most deprived areas [31]; resulting in the heightened relative COVID-19 hospitalisation and COVID-19 death rates we observed.

Nonetheless, several other vaccine centred mechanisms may also be at play. The study conducted by Fabiani and colleagues in Italy, shows that vaccine effectiveness against SARS-CoV-2 infection and severe COVID-19 decreased in the Delta epidemic phase compared to the Alpha phase [33]. This reduction in vaccine induced protection, may have exacerbated the impact of unequal housing conditions, working conditions and comorbidities amongst vaccinated individuals living in the most deprived areas (compared to vaccinated individuals living in affluent areas), contributing to the higher inequalities in SARS-CoV-2 infection, and COVID-19 health outcomes we observed here.

Finally, the autumn season partly overlapping the high vaccination coverage period, may have also influenced SARS-CoV-2 infection incidence inequalities [34]. Specifically, the increased time spent indoors in autumn compared to summer months may have promoted household viral transmission, disproportionately affecting disadvantaged populations owing to crowded housing conditions.

Our study has several limitations. First, as we used population and deprivation index estimates based on 2011 census data, likely differing for 2021, we may have slightly over- or underestimated the true associations between SED and our study outcomes. Second, despite the fact that the deprivation index we used takes into account characteristics pertaining to education, employment status and housing conditions, there may be other important deprivation components not captured by the index. Third, our study did not include periods before the vaccine rollout in order to assess the overall impact of vaccination on socioeconomic inequalities in COVID-19 [30], nor periods with Omicron variant predominance where socioeconomic inequalities in COVID-19 may have evolved differently [35]. Finally, we lacked information on co-morbidities and vaccination data geolocated at the census block level, which could provide further insights into the associations we found.

## Conclusions

In our study focussing on urban Italy, we found patterns of increasing socioeconomic inequalities in SARS-CoV-2 infection and COVID-19-related hospitalisation and death, between periods of increasing COVID-19 vaccination coverage, affecting most those residing in the most deprived areas. These inequalities likely stem from endemic inequalities in the social determinants in health and evolved in response to socioeconomic inequalities in COVID-19 vaccination coverage, vaccine effectiveness patterns, the predominance of different SARS-CoV-2 variants and the gradual suspension of restriction measures; differentially impacting socioeconomic groups over time. Our findings indicate that deprived populations in urban Italy should receive stronger attention in future pandemic preparedness plans to respond to COVID-19 and potentially other respiratory diseases, in particular during the mass vaccination roll out phases with gradual lifting of PHSMs. In addition, considering socioeconomic inequality temporal dynamics, especially over fine-scale geographic units as presented here, may support future pandemic responses in promoting health equity through the design and deployment of targeted and timely immunisation strategies, non-pharmaceutical interventions and ad hoc social and economic policies. Ultimately, we posit that enhancing pandemic preparedness in the future to a point of achieving maximum health equity necessitates addressing health inequalities through long-term solutions, focusing on tackling the underlying social determinants of health [3].

### Supplementary Information

Below is the link to the electronic supplementary material.

Supplementary file1 (DOCX 96 KB)

## Data Availability

Data generated and analyzed for the current study are not publicly available.
