# Supplementary material for: Socioeconomic Inequalities in SARS-CoV-2 Infection and COVID-19 Health Outcomes in Urban Italy During the COVID-19 Vaccine Rollout, January–November 2021
Source: J Urban Health. 2024 Mar 18;101(2):289–99. doi: 10.1007/s11524-024-00844-0 (PMC11052739; doi:10.1007/s11524-024-00844-0)
Supplement: Supplementary file 1 — Supplementary file1 (DOCX 96 KB) [file 11524_2024_844_MOESM1_ESM.docx]

**Supplementary material**

**Table S1**: Age adjusted cumulative incidence rates of SARS-CoV-2 infection, COVID-19 hospitalisation, and COVID-19 death per 1,000,000 person-days, by census block SED terciles in urban Italy, for three consecutive periods of different COVID-19 vaccination coverage. Low coverage (0 to 10%) / January 1 – March 24, 2021; intermediate coverage (>10 to 60%) / March 25 – July 25, 2021; high coverage (>60 to 74%) / July 26 – November 4, 2021, with at least one vaccine dose. D1, D2 and D3 correspond to the least, moderately and most deprived census block areas respectively. Age standardization was conducted using the revised European Standard Population (EUROSTAT).

|  | Low vaccination  coverage | | Intermediate vaccination  coverage | | | High vaccination  coverage | |
| --- | --- | --- | --- | --- | --- | --- | --- |
| **SARS-CoV-2 infections** | | |  | | |  | |
| Area ID | Number | AAR | Number | | AAR | Number | AAR |
| D1 | 86,981 | 243.9 | 52,767 | | 103.5 | 24,139 | 58.4 |
| D2 | 137,693 | 266.7 | 87,134 | | 116.7 | 40,395 | 66.2 |
| D3 | 113,200 | 269.9 | 77,707 | | 127.8 | 38,261 | 76.3 |
| **COVID-19 hospitalisations** | | | | | |  | |
| Area ID | Number | AAR | Number | AAR | | Number | AAR |
| D1 | 8227 | 20.2 | 4612 | 8.0 | | 1585 | 3.3 |
| D2 | 12,519 | 23.4 | 7594 | 9.8 | | 2809 | 4.4 |
| D3 | 11,753 | 27.9 | 7320 | 12.0 | | 3140 | 6.2 |
| **COVID-19 deaths** | | |  | | |  | |
| Area ID | Number | AAR | Number | AAR | | Number | AAR |
| D1 | 2303 | 5.3 | 924 | 1.5 | | 289 | 0.6 |
| D2 | 3367 | 6.1 | 1542 | 1.9 | | 505 | 0.8 |
| D3 | 3194 | 7.4 | 1448 | 2.3 | | 481 | 0.9 |

**
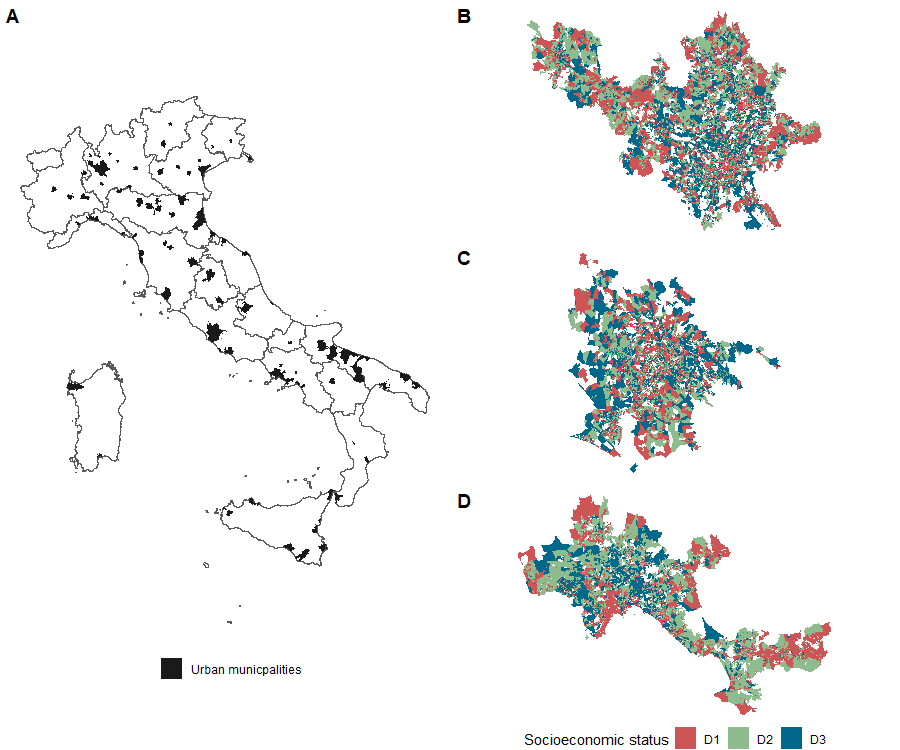
**

**Figure S1**. Geographical distribution of urban municipalities (LAU level 2), Italy, 2023 (A); Census block geographical distribution by census block socioeconomic deprivation status in the cities (i.e. ≥1 urban municipalities) of Milan (B), Rome (C), Naples (D).

**Table S2**. Fully adjusted and unadjusted incidence rate ratios (IRR) of SARS-CoV-2 infection (A), COVID-19 hospitalization (B), and COVID-19 death (C), by census block socioeconomic deprivation status in urban Italy, for three consecutive periods of different COVID-19 vaccination coverage. Low coverage (0 to 10%) / January 1 – March 24, 2021; intermediate coverage (>10 to 60%) / March 25 – July 25, 2021; high coverage (>60 to 74%) / July 26 – November 4, 2021, with at least one vaccine dose. D1, D2 and D3 correspond to the least, moderately and most deprived census block areas respectively. The fully adjusted models are adjusted for sex, NUTS1 level areas and the interaction between vaccination coverage period and age; with an interaction effect for vaccination coverage period on socioeoconomic deprivation.

|  | Low vaccination coverage | Intermediate vaccination coverage | High vaccination coverage | Unadjusted |
| --- | --- | --- | --- | --- |
| **Infection IRR [95%CI]** |  |  |  |  |
| D1 | Ref | Ref | Ref | Ref |
| D2 | 1.06 [1.00-1.12] | 1.10 [1.03-1.17] | 1.11 [1.04-1.18] | 1.07 [1.00-1.15] |
| D3 | 1.09 [1.03-1.15] | 1.22 [1.15-1.29] | 1.28 [1.21-1.37] | 1.14 [1.07-1.23] |
| **Hospitalisation IRR [95%CI]** |  |  |  |  |
| D1 | Ref | Ref | Ref | Ref |
| D2 | 1.17 [1.08-1.28] | 1.19 [1.09-1.30] | 1.32 [1.19-1.47] | 1.14 [1.01-1.30] |
| D3 | 1.48 [1.36-1.61] | 1.53 [1.41-1.67] | 2.02 [1.82-2.25] | 1.43 [1.26-1.63] |
| **Death IRR [95%CI]** |  |  |  |  |
| D1 | Ref | Ref | Ref | Ref |
| D2 | 1.14 [1.00-1.31] | 1.35 [1.15-1.57] | 1.37 [1.11-1.69] | 1.13 [0.86-1.48] |
| D3 | 1.57 [1.36-1.80] | 1.68 [1.44-1.97] | 1.89 [1.53-2.34] | 1.43 [1.09-1.88] |

**Table S3.** Sex stratified incidence rate ratios (IRR) of COVID-19 infection (A), hospitalization (B), and death (C), by census block socioeconomic deprivation status terciles in urban Italy, for three consecutive periods of different COVID-19 vaccination coverage. Low coverage (0 to 10%) / January 1 – March 24, 2021; intermediate coverage (>10 to 60%) / March 25 – July 25, 2021; high coverage (>60 to 74%) / July 26 – November 4, 2021, with at least one vaccine dose. D1, D2 and D3 correspond to the least, moderately and most deprived census block areas respectively. The models are adjusted for NUTS1 level areas, and the interaction between vaccination coverage period and age; with an interaction effect for vaccination coverage period on SED.

|  |  | Low vaccination coverage | Intermediate vaccination coverage | High vaccination coverage |
| --- | --- | --- | --- | --- |
|  | Infection IRR [95%CI] | |  |  |
| Females | D1 | Ref | Ref | Ref |
|  | D2 | 1.07 [0.99-1.16] | 1.11 [1.02-1.21] | 1.12 [1.02-1.22] |
|  | D3 | 1.12 [1.03-1.22] | 1.25 [1.15-1.36] | 1.32 [1.21-1.44] |
| Males | D1 | Ref | Ref | Ref |
|  | D2 | 1.05 [0.96-1.14] | 1.08 [1.00-1.18] | 1.10 [1.00-1.20] |
|  | D3 | 1.06 [0.97-1.15] | 1.18 [1.09-1.29] | 1.25 [1.14-1.37] |
|  | Hospitalisation IRR [95%CI] | |  |  |
| Females | D1 | Ref | Ref | Ref |
|  | D2 | 1.26 [1.12-1.41] | 1.27 [1.12-1.43] | 1.38 [1.19-1.60] |
|  | D3 | 1.67 [1.48-1.87] | 1.63 [1.45-1.84] | 2.14 [1.85-2.49] |
| Males | D1 | Ref | Ref | Ref |
|  | D2 | 1.11 [1.00-1.24] | 1.13 [1.01-1.26] | 1.27 [1.11-1.46] |
|  | D3 | 1.33 [1.19-1.47] | 1.45 [1.30-1.62] | 1.89 [1.65-2.17] |
|  | Death IRR [95%CI] |  |  |  |
| Females | D1 | Ref | Ref | Ref |
|  | D2 | 1.09 [0.89-1.33] | 1.45 [1.15-1.83] | 1.51 [1.11-2.06] |
|  | D3 | 1.55 [1.27-1.90] | 1.80 [1.42-2.27] | 1.82 [1.33-2.48] |
| Males | D1 | Ref | Ref | Ref |
|  | D2 | 1.19 [1.00-1.43] | 1.27 [1.04-1.56] | 1.26 [0.96-1.67] |
|  | D3 | 1.57 [1.31-1.88] | 1.60 [1.30-1.96] | 1.93 [1.46-2.56] |

**Table S4.** Age stratified incidence rate ratios (IRR) of SARS-CoV-2 infection (A), COVID-19 hospitalization (B), and COVID-19 death (C), by census block socioeconomic deprivation status terciles in urban Italy, for three consecutive periods of different COVID-19 vaccination coverage: low coverage (0 to 10%) / January 1 – March 24, 2021; intermediate coverage (>10 to 60%) / March 25 – July 25, 2021; high coverage (>60 to 74%) / July 26 – November 4, 2021, with at least one vaccine dose. D1, D2 and D3 correspond to the least, moderately and most deprived census block areas respectively. The models are adjusted for sex, NUTS1 level areas, and the interaction between vaccination coverage period and age; with an interaction effect for vaccination coverage period on SED.

|  |  | Low vaccination coverage | Intermediate vaccination coverage | High vaccination coverage |
| --- | --- | --- | --- | --- |
|  | Infection IRR [95%CI] |  |  |  |
| 20-59 years | D1 | Ref | Ref | Ref |
|  | D2 | 1.03 [0.96-1.11] | 1.05 [0.98-1.13] | 1.09 [1.01-1.17] |
|  | D3 | 1.03 [0.96-1.10] | 1.14 [1.06-1.22] | 1.27 [1.18-1.37] |
| ≥ 60 years | D1 | Ref | Ref | Ref |
|  | D2 | 1.11 [1.00-1.24] | 1.21 [1.08-1.34] | 1.15 [1.03-1.29] |
|  | D3 | 1.22 [1.10-1.35] | 1.40 [1.26-1.56] | 1.32 [1.18-1.48] |
|  | Hospitalisation IRR [95%CI] |  |  |  |
| 20-59 years | D1 | Ref | Ref | Ref |
|  | D2 | 1.18 [1.05-1.33] | 1.21 [1.07-1.36] | 1.36 [1.17-1.58] |
|  | D3 | 1.51 [1.34-1.70] | 1.59 [1.41-1.80] | 2.27 [1.96-2.63] |
| ≥ 60 years | D1 | Ref | Ref | Ref |
|  | D2 | 1.17 [1.05-1.31] | 1.19 [1.05-1.33] | 1.30 [1.12-1.49] |
|  | D3 | 1.45 [1.29-1.62] | 1.47 [1.31-1.65] | 1.74 [1.51-2.01] |
|  | Death IRR [95%CI] |  |  |  |
| 20-59 years | D1 | Ref | Ref | Ref |
|  | D2 | 1.13 [0.82-1.56] | 1.97 [1.32-2.93] | 1.39 [0.78-2.45] |
|  | D3 | 1.86 [1.36-2.55] | 2.47 [1.66-3.68] | 2.26 [1.31-3.92] |
| ≥ 60 years | D1 | Ref | Ref | Ref |
|  | D2 | 1.16 [1.00-1.34] | 1.27 [1.08-1.50] | 1.38 [1.11-1.72] |
|  | D3 | 1.51 [1.31-1.75] | 1.59 [1.35-1.88] | 1.82 [1.46-2.28] |

**Table S5.** Sensitivity analysis for different vaccination coverage periods. Fully adjusted and unadjusted incidence rate ratios (IRR) of SARS-CoV-2 infection (A), COVID-19 hospitalization (B), and COVID-19 death (C), by census block Socioeconomic Status Index tertiles in urban Italy, for three consecutive periods of different COVID-19 vaccination coverage: period 1 (0 to 20%) / January 1 – April 21, 2021; period 2 (>20 to 65%) /April 22 –August 16, 2021; period 3 (>65 to 74%) / August 17 – November 4, 2021, with at least one vaccine dose. The models are adjusted for sex, NUTS1 level areas, and the interaction between vaccination coverage period and age; with an interaction effect for vaccination coverage period on SED.

|  | Low vaccination  coverage | Intermediate  vaccination coverage | High vaccination  coverage | Unadjusted |
| --- | --- | --- | --- | --- |
| Infection IRR [95%CI] | |  |  |  |
| D1 | Ref | Ref | Ref | Ref |
| D2 | 1.07 [1.01-1.13] | 1.07 [1.01-1.14] | 1.16 [1.09-1.23] | 1.07 [0.98-1.18] |
| D3 | 1.11 [1.06-1.18] | 1.22 [1.15-1.29] | 1.35 [1.27-1.43] | 1.14 [1.04-1.25] |
| Hospitalisation IRR [95%CI] | |  |  |  |
| D1 | Ref | Ref | Ref | Ref |
| D2 | 1.20 [1.11-1.29] | 1.19 [1.08-1.31] | 1.31 [1.17-1.46] | 1.14 [0.99-1.32] |
| D3 | 1.50 [1.39-1.62] | 1.67 [1.52-1.83] | 2.01 [1.80-2.25] | 1.44 [1.25-1.65] |
| Death IRR [95%CI] | |  |  |  |
| D1 | Ref | Ref | Ref | Ref |
| D2 | 1.18 [1.04-1.33] | 1.43 [1.19-1.73] | 1.36 [1.10-1.69] | 1.13 [0.85-1.51] |
| D3 | 1.61 [1.43-1.82] | 1.73 [1.43-2.09] | 1.83 [1.48-2.28] | 1.44 [1.07-1.92] |
